# Dr. Kinglake, on Admissible Criticism

**Published:** 1809-02

**Authors:** Robert Kinglake

**Affiliations:** Taunton


THE
Medical and Physical Journal.

VOL. XXI.] FEBRUARY, 1809. [NO. 120.

*Printed for R. PHILLIPS, by W. Thorne, Red Lion Court, Fleet Street, London.*

### *To the Editors of the Medical and Physical Journal.*

GENTLEMEN,

THE charge of hyper-criticism, imputed by "Scriblerus" to Dr. Beddoes in your last Journal, does not appear to be well-founded. Not only in strictness of etymology, but in correctness of phraseology, Dr. Beddoes seems to be perfectly warranted in resisting the gross defect obviously implied by "Morbid Anatomy." This phrase, whatever may be its popular acceptation, conveys an idea of imperfection in the mode of anatomising. The signification of morbid, as an adjective, cannot be applied to any substantive expressive of an object in which imperfection is not necessarily involved, without literally avowing it to be defective.

The respectable author of a celebrated work, entitled "Morbid Anatomy," has probably been betrayed into the adoption of this exceptionable phrase, rather from its unobjected currency, than from the exercise of his own critical judgment on its propriety.

No person, perhaps, is more competent than himself, both to think and speak correctly on anatomical subjects, and his well known candour will probably not deem "hyper-critical" the objection which has been taken against an expression sanctioned by his name.

Verbal criticism is always likely to have ample justice done it by the ingenuity of Dr. Beddoes; and his laudable anxiety for correctness, renders him unsparingly rigorous for its establishment.

*Pathological anatomy,* (anatomie pathologique) adopted by the French, and proposed by Dr. Beddoes, as a substitute for "Morbid Anatomy," is undoubtedly preferable, and conveys a tolerably accurate idea of what is intended; but it has too much of technical Greek about it, and too much of the ancient as well as the modern fashion, to refer to that dead language for terms which might, perhaps, be always as appropriately and more intelligibly furnished by by the native tongue. It can hardly be disputed, that in the investigation of disease, the manner in which it is done, should be described by a participle perfect, explaining the affection or condition of its relative object. This significant expression would appear to be sufficiently afforded by the phrase *anatomised disease*. This designation cannot be liable to a doubtful meaning, it very directly shews that disease is the object of anatomical research, and that the anatomy employed, is not *morbid* or imperfect.

If I mistake not, the German language has a compound word much more descriptive of pathological anatomy, or anatomised disease, than either *krankhafte Zergliederungskunst*, or *krankhafter Bau Zergliederung*, which is, *krankhaits—Zergliederung*, (anatomy of disease.)

Next in importance to knowing things accurately, is the use of such apposite words as will convey a precise notion of them; without which, there can be no instructive correspondence subsisting between the one and the other. To imagine a thing correctly, and to word it inaccurately, is to erect an insuperable stumbling block between thought and expression.

ROBERT KINGLAKE.

*Taunton, Dec.* 20, 1808.

P. S. Jan. 3, 1809. With heartfelt sorrow, I have just learnt the death of the truly estimable Dr. Beddoes. My friendship for him, and persuasion of his philological accuracy on the subject of the above remarks, induced me to offer them to the public. Had the intelligent author of the objection to the incorrect phrase referred to, lived to have extended his comments on it, the subject would have been likely soon to have obtained all the explanation it may require. As it was my intention to have published the above observations before the regretted event of Dr. Beddoes's death had occurred, I feel it as a sort of tribute due to his respected memory not now to withhold them; and it would appear but justice to add, that science has lost in the death of Dr. Beddoes one of the most original, acute, and liberal cultivators of its best interests that the present age affords.

---

Our respect for Dr. KINGLAKE, and the only tribute we can pay to the *illustrious dead*, make us ready to give insertion to the above; but we hope the Controversy will end here. Our readers will judge for themselves, having the whole question before them in the present and two preceding numbers. We are favoured by one of the first philosophers philosophers of the age with the following extract from a letter, written by Dr. Beddoes's own hand two days before his death. Ed.

"Quietly and stilly have I put forth a bit of solid pathological doctrine, drawn from no superficial sources. What I refer to is in the Medical and Physical Journal in the two last months; it is briefly this, that in some circumstances a man shall die apoplectic, or with inflamed head, and shall have an inflammation in his stomach, sometimes of standing, at others arising in a few hours; and whichever the duration, no pain be felt in the stomach, and indeed the head will go on long too."

